# What is the relationship between population health and voting patterns: an ecological study in England

**DOI:** 10.1136/bmjresp-2025-003526

**Published:** 2025-10-14

**Authors:** Anthony A Laverty, Nicholas S Hopkinson

**Affiliations:** 1 Department of Primary Care and Public Health, Imperial College London School of Public Health, London, UK; 2 National Heart and Lung Institute, Imperial College London, London, UK

**Keywords:** Clinical Epidemiology, COPD epidemiology, Asthma Epidemiology

## Abstract

**Background:**

Health is a fundamental issue in politics and an area where governments hold significant levers of influence. Countries in Europe have seen increased support for populist political parties, with some evidence linking support for these parties to health metrics. We aimed to establish if there is an association between health metrics and patterns of voting in England, particularly in relation to a recently established political party, Reform UK, in the 2024 general election.

**Methods:**

We conducted a cross sectional ecological study with data from all constituencies in England (n=543). We conducted Pearson correlations and linear regression between the proportion of eligible votes for Reform UK and estimated prevalence of 20 common non-communicable diseases, including obesity, chronic obstructive pulmonary disease (COPD), asthma, type 2 diabetes and depression.

**Results:**

Constituencies electing Reform members of parliament (MPs) (n=5/543) had the highest average prevalence of asthma (7.44%) and COPD (2.85%). Across the country, adjusting for age, sex and deprivation, a 10% increase in the party’s vote share was associated with a +0.261% (95% CI 0.213% to 0.309%) prevalence of COPD, a +0.113% (95% CI 0.026% to 0.201%) prevalence of asthma and a +1.479% (95% CI 1.239% to 1.720%) increase in obesity prevalence.

**Conclusions:**

At a constituency level, poor health, in particular conditions associated with breathlessness, was associated with a greater proportion of votes for Reform UK.

**WHAT IS ALREADY KNOWN ON THIS TOPIC**

- Countries in Europe have seen a rise in support for populist parties.
- While the reasons behind this are complex, research in Italy and the US has linked support for such parties to poorer health outcomes.
- No research has examined the issue of health in relation to voting for Reform UK in England.

**WHAT THIS STUDY ADDS**

- We compared estimated average prevalences of 20 common non-communicable diseases between areas (n=5/543) returning a Reform UK member of parliament (MP) in the 2024 general election and areas returning MPs from a different party. We also assessed relationships between the proportion of Reform UK votes and these health metrics.
- Constituencies electing Reform MPs had the highest average prevalence of 15/20 conditions, including asthma and chronic obstructive pulmonary disease (COPD).
- Linear regression controlled for age, sex and deprivation found that increases in the vote share for Reform UK vote was associated with poorer health metrics, including asthma, COPD and obesity.

**HOW THIS STUDY MIGHT AFFECT RESEARCH, PRACTICE OR POLICY**

- This ecological study suggests a link between poorer health status and votes for Reform UK.
- Further research could usefully work to understand possible mechanisms behind this, and this study highlights the importance of improving health across the whole of England.

## Introduction

 Health is a fundamental issue in politics, essential both in relation to the wellbeing of citizens and for a nation’s economic productivity. Decisions by policy makers influence both the provision of healthcare services, and the upstream social and commercial determinants of health, which include clean air, transport, food, housing and employment. Lung health, in particular, is influenced by health inequality.^1^,^5^ At the same time, experience of good or poor health, and interactions with the healthcare system, may influence voters’ perceptions of incumbent political parties and the alternatives being offered to them. It is therefore to be expected that there will be interactions between health and public opinion, in particular, the way that opinion is expressed through voting in elections.

In the July 2024 UK general election, Reform UK, a political party founded in 2018, secured 14% of votes, winning five out of the 543 English constituencies, and therefore had five members of the UK parliament (MPs). More recently, in May 2025, the party has won a substantial number of council seats in local authority elections. Many countries in Europe and elsewhere have seen a rise in similar populist parties and there is evidence that support for them is linked to both health and satisfaction with healthcare services.^6^,^10^ We therefore wished to establish whether there was a relation between constituency level health metrics in England and voting patterns, particularly in relation to this new party.

## Methods

Data from the 2024 general election, including the size of the electorate, number of valid votes and votes per political party, came from the House of Commons Library.^11^ Our analyses are all at the constituency level for England only. We used two measures of the strength of support for Reform UK: (1) constituencies in England returning a Reform MP compared with those electing a different political party and (2) the proportion of votes for Reform across all constituencies.

Health outcomes were the prevalence of 20 common health conditions collated by the House of Commons Library, based on the NHS Quality and Outcomes Framework data from 2022–23: asthma; atrial fibrillation; cancer diagnosis (any); chronic kidney disease; chronic obstructive pulmonary disease (COPD); coronary heart disease; dementia; depression; type 2 diabetes; epilepsy; heart failure; hypertension; learning disabilities; non-diabetic hyperglycaemia; obesity; osteoporosis; peripheral arterial disease; rheumatoid arthritis; schizophrenia, bipolar disorder and psychoses; and stroke/transient ischaemic attack (combined).^12^ We took age and sex data of resident populations from the Office for National Statistics population estimates for 2022, as well as the Index of Multiple Deprivation data, which compares constituencies on their relative deprivation, divided into five groups for analyses.^12 13^

We first compared the average prevalences of each health condition according to the MP returned in the general election (Labour Party, Conservative and Unionist Party, Liberal Democrats, Green Party and Reform UK). We used Pearson correlation coefficients to investigate relationships between Reform UK vote share and health metrics. Finally, we conducted separate linear regression models for each health metric, controlling for age, sex and deprivation. We did not account or correct for multiple testing as the illnesses studied here are not independent of each other.

## Results

Of 543 constituencies in England, Labour won 347, the Conservatives 116, the Liberal Democrats 65, the Green Party four and Reform UK won five seats (total=537) in the 2024 general election. We excluded constituencies returning an independent MP (n=5), as well as the constituency of the Speaker of the House (n=1), as by convention the main parties did not put up a candidate against him.

Three of the five areas (60%) returning a Reform UK MP were in the most deprived fifth of the country, compared with 103 (29.7%) of Labour constituencies (table 1). Reform UK areas also had the highest proportions of residents aged >65 years (23.8% vs 17.1% for Labour and 23.2% for the Conservatives).

**Table 1:** Age, sex and deprivation breakdown of constituencies by winning party in 2024 UK general election

Winning party 2024	No of constituencies	No (%) in most deprived fifth*	Mean (SD) % aged >65 years	Mean (SD) % men
Labour	347	103 (29.7)	17.1 (0.05)	49.0 (0.73)
Conservative	116	0	23.2 (0.04)	48.8 (0.58)
Liberal Dem	65	0	22.1 (0.05)	48.8 (0.51)
The Green Party	4	0	19.5 (0.10)	49.3 (0.53)
Reform UK	5	3 (60.0)	23.8 (0.05)	48.7 (0.40)

* As measured by the Index of Multiple Deprivation.

**Figure 1:**
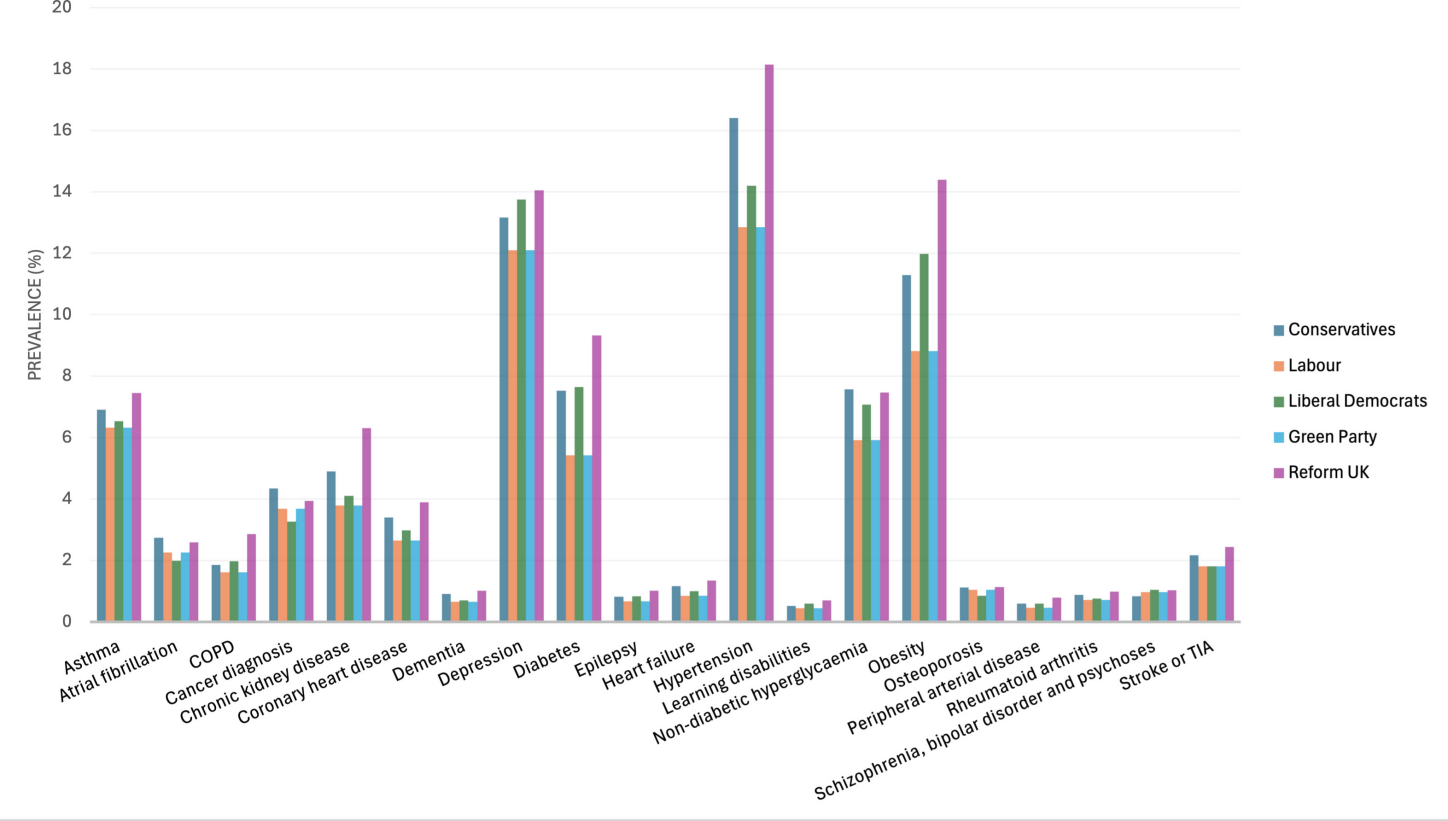
Average prevalence of common conditions by winning party at 2024 UK general election. COPD, chronic obstructive pulmonary disease; TIA, transient ischaemic attack.

The five areas that returned a Reform UK MP had the highest average prevalence of 15 out of 20 health conditions: asthma; COPD; chronic kidney disease; coronary heart disease; dementia; depression; diabetes; epilepsy; heart failure; hypertension; learning disabilities; obesity; peripheral arterial disease; rheumatoid arthritis;and stroke or transient ischaemic attack (figure 1 and table 2). For example, Reform UK constituencies had an average asthma prevalence of 7.44% and an average COPD prevalence of 2.85% compared with 6.58% and 1.99% for Labour areas. Reform UK areas had an average prevalence of coronary heart disease of 3.90% compared with 2.98% in Conservative areas, and an average depression prevalence of 14.05% compared with 12.84% in Liberal Democrat areas.

**Table 2:** Mean prevalence of health conditions by winning party at 2024 UK general election

Condition	Mean prevalence %				
	Labour	Conservative	Lib Dem	Green	Reform UK
Asthma	6.54	6.90	6.84	6.33	7.44
Atrial fibrillation (irregular heart rate)	2.00	2.74	2.65	2.27	2.59
COPD	1.97	1.85	1.63	1.62	2.85
Cancer diagnosis since 2003	3.27	4.35	4.31	3.69	3.95
Chronic kidney disease	4.10	4.89	4.47	3.78	6.31
Coronary heart disease	2.98	3.41	3.11	2.65	3.90
Dementia	0.70	0.92	0.87	0.66	1.01
Depression	13.75	13.16	12.84	12.10	14.05
Diabetes	7.65	7.52	6.55	5.43	9.33
Epilepsy	0.83	0.81	0.78	0.67	1.02
Heart Failure	0.99	1.16	1.02	0.85	1.34
High blood pressure (hypertension)	14.21	16.41	15.02	12.85	18.15
Learning disabilities	0.59	0.52	0.50	0.44	0.70
Non-diabetic hyperglycaemia	7.07	7.56	7.23	5.92	7.46
Obesity	11.98	11.29	9.92	8.82	14.40
Osteoporosis	0.86	1.13	1.23	1.05	1.14
Peripheral arterial disease	0.60	0.59	0.55	0.47	0.79
Rheumatoid arthritis	0.75	0.89	0.79	0.71	0.98
Schizophrenia, bipolar disorder and psychoses	1.05	0.84	0.87	0.97	1.03
Stroke or transient ischaemic attack	1.81	2.17	2.07	1.80	2.43

COPD, chronic obstructive pulmonary disease .

**Figure 2:**
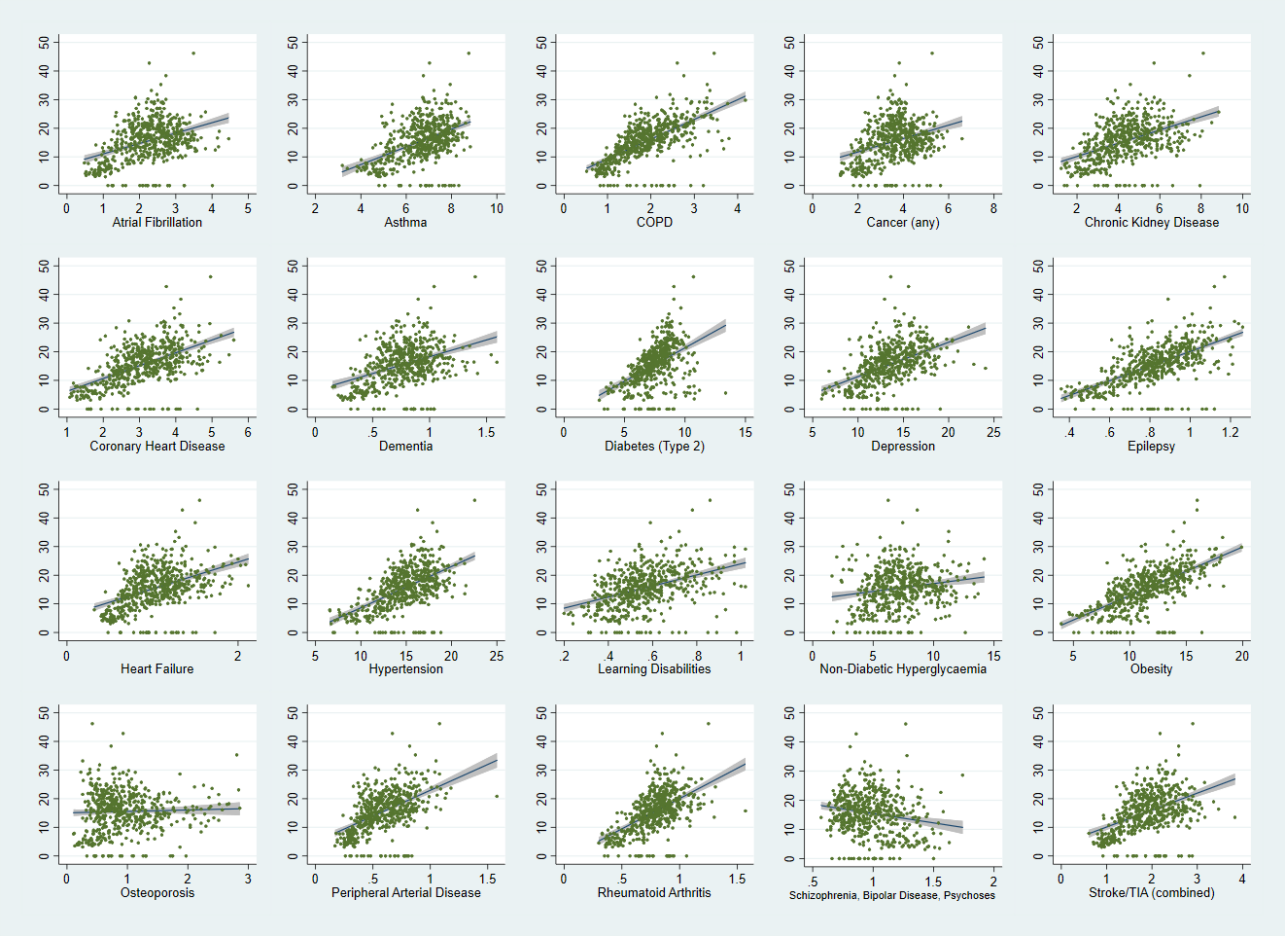
Correlations between Reform UK vote proportions and selected non-communicable diseases. X-axis represents prevalance of conditions; y-axis represents proportion of Reform UK votes.

Vote share for Reform UK in individual constituencies ranged from 0% to 46.2%, with positive correlations between Reform UK vote share and the prevalence of 19/20 health measures (table 3 and figure 2). Seven of these were strong correlations (>0.5) and 10 were moderately sized (ie, between 0.3 and 0.49). The strongest correlations were for obesity (0.654), COPD (0.647) and epilepsy (0.632) (all p<0.001).

**Table 3:** Unadjusted Pearson correlation coefficients between Reform UK vote share and health outcomes at the constituency level in England (sorted largest to smallest)

Condition	Correlation	P value
Obesity	0.654	<0.001
Chronic obstructive pulmonary disease	0.647	<0.001
Epilepsy	0.632	<0.001
Hypertension	0.577	<0.001
Rheumatoid arthritis	0.548	<0.001
Coronary heart disease	0.537	<0.001
Peripheral arterial disease	0.520	<0.001
Depression	0.479	<0.001
Asthma	0.471	<0.001
Diabetes	0.468	<0.001
Stroke or transient ischaemic attack	0.457	<0.001
Chronic kidney disease	0.448	<0.001
Heart failure	0.442	<0.001
Learning disability	0.405	<0.001
Dementia	0.388	<0.001
Atrial fibrillation	0.382	<0.001
Cancer	0.323	<0.001
Non-diabetic hyperglycaemia	0.174	<0.001
Osteoporosis	0.036	0.3988
Schizophrenia, bipolar disorder and psychoses	−0.187	<0.001

After controlling for age, sex and Index of Multiple Deprivation, there were statistically significant (p<0.05) positive relationships between Reform UK vote share and the prevalence of 15/20 conditions (table 4). The largest association was for obesity, where a 10% increase in Reform UK vote share was associated with a +1.479% (95% CI 1.239% to 1.720%) increase in obesity prevalence. For each 10% increase in Reform UK vote share, there was a +0.261% (95% CI 0.213% to 0.309%) higher prevalence of COPD, a +0.113% (0.026% to 0.201%) greater prevalence of asthma, and a +0.980% (0.654% to 1.306%) increased prevalence of depression.

**Table 4:** Relationship between Reform UK vote share and prevalence of health conditions

Condition	Coefficient	P value	Lower CI	Upper CI
Asthma	0.113	0.011	0.026	0.201
Atrial fibrillation	0.026	0.061	−0.001	0.053
Cancer	0.029	0.190	−0.014	0.071
Chronic kidney disease	0.251	<0.001	0.120	0.381
Chronic obstructive pulmonary disease	0.261	<0.001	0.213	0.309
Coronary heart disease	0.119	<0.001	0.072	0.166
Dementia	0.023	0.002	0.008	0.037
Depression	0.980	<0.001	0.654	1.306
Diabetes	0.459	<0.001	0.305	0.614
Epilepsy	0.068	<0.001	0.054	0.082
Heart failure	0.030	0.039	0.002	0.059
Hypertension	0.824	<0.001	0.677	0.970
Learning disability	0.025	0.002	0.009	0.041
Non-diabetic hyperglycaemia	−0.098	0.523	−0.400	0.204
Obesity	1.479	<0.001	1.239	1.720
Osteoporosis	−0.056	0.109	−0.125	0.013
Peripheral arterial disease	0.027	0.001	0.011	0.043
Rheumatoid arthritis	0.057	<0.001	0.043	0.072
Schizophrenia, bipolar disorder and psychoses	−0.093	<0.001	−0.113	−0.073
Stroke or transient ischaemic attack	0.019	0.155	−0.007	0.046

Results from separate linear regressions, adjusted for percentage of local area aged >65 years; sex; and Index of Multiple Deprivation in five groups. Results shown for Reform UK vote share only and represent changes in percentage of population with health conditions per 10% increase in Reform UK vote share.

## Discussion

The main finding of our analysis was an association between poor health metrics at a constituency level and votes for Reform UK. The five constituencies where the party won a seat had the highest average prevalence of 15 out of 20 long term health conditions, and across the country there were positive correlations between the percentage vote for Reform UK and the constituency prevalence of 19out of 20 conditions. These relationships persisted when adjusted for constituency demographics and levels of deprivation.

The results are consistent with work showing a relationship between poor healthcare measures and Republican voting in the US^6^ and data from Italy linking dissatisfaction with public services and voting for the far right.^7^ In the UK, closure of local healthcare facilities has been shown to reduce reported patient satisfaction and increase support for populist right parties.^8^

Lung health is particularly influenced by health inequality,^1^,^5^ and conditions causing breathlessness (obesity, COPD as well as asthma and cardiac disease) appear in turn to be linked to voting patterns. Different factors are potentially behind links between health and voting patterns in other countries, and these likely include the structural determinants of health, such as medical care and housing. In the UK, the introduction of austerity policies, aggravated by the effects of the COVID-19 pandemic, has contributed to the fact that many people with long term lung conditions are missing out on basic aspects of care, which may fuel frustration with the status quo.^1 2 4^ Living in a home that is cold and/or damp is associated with an increased risk of acute exacerbations and hospitalisation,^4^ so poor housing will also interact with health experience.

Three of the five Reform UK voting constituencies are coastal. The 2021 Chief Medical Officer’s report^14^ highlighted the particular health issues in coastal areas, due to greater ill health in older poorer populations with more long term conditions, while healthcare provision is lower than in other parts of the country, a specific manifestation of the inverse care law.^15^

Depression can be characterised by negative feelings about the self, world and future,^16^ as well as a reduction in the sense of being in control of one’s life.^17 18^ A recent US study found an association between depression and misperception as to the legitimacy of election results.^19^ Experience of mental as well as physical health issues may therefore influence decisions around voting and support for populist parties.

### Limitations

We acknowledge some limitations in our study. Election data came from 2024 while health indicators came from 2022–23 (the latest available for these geographies). Both health and political sentiment are influenced by long term trends which are not captured in this cross sectional study. Approximately 90% of constituency boundaries were changed for the 2024 election, so longitudinal analyses are difficult to conduct. Finally, as analyses are at aggregate geographies, the ecological fallacy is possible here and only limited conclusions about individual behaviour can be drawn from average population characteristics. Future work, both quantitative and qualitative, should address these issues and potential mechanisms.

## Conclusions

Government holds many levers that can influence health, including decisions around spending on healthcare and other aspects of infrastructure, such as transport and education, redistributive policies to reduce poverty, legislation to address commercial determinants of health, as well as social and community services to support people living with long term health conditions.^3^ While these analyses are ecological, they support a link between poorer health and increased votes for Reform UK. This suggests a useful synergy across the political spectrum. For Reform UK policy makers, they demonstrate that there are profound health issues in their constituencies which should be addressed. For those elsewhere in the political spectrum, these results should provide a further incentive to take steps to improve public health and reduce inequalities.^20^,^23^

## Data Availability

Data are available in a public, open access repository.
